# The Contribution of Low-Frequency and Rare Coding Variation to Susceptibility to Type 2 Diabetes

**DOI:** 10.1007/s11892-019-1142-5

**Published:** 2019-04-08

**Authors:** Jason Flannick

**Affiliations:** 1 Division of Genetics and Genomics, Boston Children’s Hospital, Boston, MA USA; 2 Department of Pediatrics, Harvard Medical School, Boston, MA 02115 USA; 3 Programs in Medical and Population Genetics and Metabolism, Broad Institute of Harvard and MIT, Cambridge, MA 02142 USA

**Keywords:** Rare variants, Coding variants, Exome, Sequencing, GWAS, RVAS, Genetic architecture

## Abstract

**Purpose of Review:**

Soon after the first genome-wide association study (GWAS) for type 2 diabetes (T2D) was published, it was hypothesized that rare and low-frequency variants might explain a substantial proportion of disease risk. Rare coding variants in particular were emphasized given their large expected role in disease. This review summarizes the extent to which recent T2D genetic studies provide evidence for or against this hypothesis.

**Recent Findings:**

Following a comprehensive study of T2D genetic architecture using three sequencing and genotyping technologies, four even larger studies have provided a yet higher resolution view of the role of rare and low-frequency coding variation in T2D susceptibility.

**Summary:**

Empirical evidence strongly suggests that common regulatory variants are the dominant contributor to T2D heritability. However, rare coding variants may nonetheless be pervasive across T2D-relevant genes. A strategy using common variants to map disease genes, and rare coding variants to link molecular gene perturbations to cellular and phenotypic effects, may be an effective means to investigate T2D pathogenesis and potential new therapies.

## Introduction

Genetic studies of complex diseases are largely motivated by two goals: to understand the heritable risk factors for disease in the population, and to identify biological processes relevant to disease pathogenesis [1]. The first goal seeks to quantify the contribution of different classes of genetic variation to disease heritability [2]. The second seeks to identify genetic “experiments of nature” that link genes or pathways to disease risk and potentially suggest new therapeutic strategies [3].

Coding variants have long been an emphasis in genetic studies for type 2 diabetes (T2D) and other complex diseases. Because they constitute the bulk of known genetic risk factors for Mendelian diseases, they have been hypothesized to contribute disproportionately to complex disease heritability [4–6]. Because their effects are usually easier to interpret than those of noncoding variants, they can lead to clear hypotheses about a disease-relevant gene and its directional relationship with disease risk (i.e., whether loss of function predisposes to or protects from disease) [5, 7]. The demonstration in 2004 that loss of function mutations in *PCSK9* lower low-density lipoprotein levels [8] and protect from coronary artery disease [9], and the successful cholesterol-lowering PCSK9 inhibitors consequently developed [10], have served as longstanding exemplars for many complex diseases.

When the first genome-wide association studies (GWAS) for T2D were published in 2007, some observers were therefore surprised that (a) most associations mapped outside of protein-coding regions of the genome [11] and (b) the identified associations explained only a relatively small portion of disease risk [2]. Early GWAS thus produced the first robust associations for T2D—a clear success [1, 12]—but in few cases provided clear insight into T2D’s genetic basis or its molecular and cellular mechanisms [5, 7, 13]. However, because GWAS directly or indirectly analyze only a limited set of common (minor allele frequency [MAF] > 5%) variants in the genome, their associations are not expected to explain all (or even most of) disease heritability, and might in fact tag disease-causal variants some distance away [2, 5].

This review will discuss how these early GWAS findings inspired a decade of studies to understand the role of low-frequency (MAF < 5%) and rare (MAF < 0.5%) coding variation in T2D susceptibility. In the past few years, a clear picture has begun to emerge as to how these variants contribute to T2D heritability and might be used to better understand T2D biology.

## Hypotheses, Conceptual Frameworks, and Experimental Approaches

Following early GWAS findings, three hypotheses (or models) emerged about the contribution of low-frequency and rare variants to the “genetic architecture” of complex diseases. First, rare variants were hypothesized to have significantly larger effects on disease risk than do common variants [5, 7, 14, 15]. Purifying natural selection might prevent strong-effect variants from becoming common in a population [5, 16, 17], which could explain the empirically modest effects (odds ratio [OR] < 1.1) on disease risk of most common variants [1, 2]. Strong-effect, low-frequency variants could be more clinically or therapeutically actionable than modest-effect common variants [3, 18].

Second, rare variants were hypothesized to explain a significant amount of disease heritability [2, 7, 13]. There are many more rare variants than common variants within the population [19, 20], and GWAS by design do not interrogate them. If rare or low-frequency variants have significantly larger effects on average than do common variants, then they in aggregate could explain much of the heritability not captured by GWAS.

Third, rare variants were hypothesized to cause some, and perhaps a significant portion of, common variant GWAS associations. By chance, it is possible that one or more disease-causal rare variants may segregate non-randomly with a common variant, creating a “synthetic association” detected by a common variant GWAS [5, 21]. If synthetic associations are commonplace, they could impact the design of “fine mapping” studies—efforts to localize a GWAS “index variant” association to a causal variant(s)—because index variants may lie significantly further from causal rare variants than they are expected to lie from causal common variants [21].

Testing these three hypotheses for T2D and other complex diseases required advances in rare variant ascertainment, genotyping, and association analysis. Foremost, rare variants can only be comprehensively ascertained through sequencing, and their large-scale study therefore required technology to advance from traditional Sanger sequencing of individual genes to cost-effective high-throughput next-generation sequencing [22]. By 2010, next-generation sequencing technologies were inexpensive enough to apply to thousands of samples at select regions of the genome, beginning with several genes [23, 24] and soon expanding to the entire exome [25, 26]. Because cost considerations limited the total size of regions sequenced, most early studies focused on protein-coding regions of the genome.

As whole-exome sequence (and later whole-genome sequence) data progressively accrued, opportunities also emerged to genotype a subset of coding variants detected by sequencing in much larger sample sizes. By 2012, enough European ancestry exomes had been sequenced to enable design of an inexpensive SNP microarray (the Illumina Exome Array) capturing (at a cost one order of magnitude less than sequencing) over 80% of MAF > 0.5% coding variants in Europeans [27]. By 2016, enough European ancestry genomes had been sequenced to enable a reference panel (compiled by the Haplotype Reference Consortium [HRC]) for high-quality imputation of MAF > 0.1% variants in European ancestry samples (provided the samples had been previously genotyped by a genome-wide SNP microarray) [28]. Exome array analysis and HRC-based imputation complement exome sequencing by trading off full variant ascertainment for increased study sample size (and therefore association power).

Exome array or HRC-based imputation studies predominantly employ traditional GWAS analyses, which test variants individually for disease association (“single-variant analysis”). By contrast, analyses of rare variants from exome sequencing studies require different approaches [25]. In the early 2010s, a significant number of methods were advanced to aggregate rare variants and test for association at the level of genes (“gene-level analysis”) [29]. The most basic methods collapse variants of similar molecular effect and test for different frequencies of variation between disease cases and controls (“burden tests”). While simple and easy to interpret, burden tests rely on judicious selection of variants included in the test: their application has therefore been aided by bioinformatic algorithms for predicting protein-damaging variants and theoretical frameworks for understanding how different variant selection strategies impact power to detect association [30, 31]. Alternatively, statistical tests can be made more robust to the inclusion of benign variants in gene-level analysis, a strategy that motivated the design of tests such as SKAT [32] and SKAT-O [33] that test for an overdispersion of variant associations within a gene, rather than simply a directional excess of variation in cases or controls. Most rare variant studies therefore apply multiple methods for gene-level analysis, which increases the number of tests performed, but because most studies have far fewer genes than variants, the study-wide multiple testing burden is ultimately reduced relative to GWAS.

By the early part of the 2010s, therefore, sequencing technologies and rare variant analysis methodologies were sufficiently advanced to begin the first empirical assessments of the role of low-frequency and rare variation in the development of T2D (Table 1).

**Table 1:** Technologies for interrogating low-frequency and rare variants

	Exome array	GWAS imputation	Whole-exome sequencing	Whole-genome sequencing
Properties				
Ascertainment	70–80% of MAF > 0.5% variants	Statistical inference of MAF > 0.1% variants (Europeans) or MAF > 1% variants (other populations)	All coding variants	All variants
Analysis	Single-variant	Single-variant	Gene-level	Single-variant, gene-level
Current T2D sample size	~ 500K	~ 1M	~ 50K	~ 3K
Contribution to testing rare and low-frequency variant hypotheses				
Large effects	Medium	Medium	Medium	Low
Missing heritability	Medium	Medium	High	Low
Synthetic associations	Low	Low	Medium	High

Four genotyping technologies and/or study designs (columns) have been used to identify low-frequency and rare variants associated with T2D. Each ascertains different variants (Ascertainment), enables different association analysis methodologies (Analysis), and has been applied to different sample sizes for T2D (Current T2D sample size). The bottom half of the table summarizes the historical contribution of each study design toward evaluating the validity of three rare variant hypotheses about the role of rare variation in T2D susceptibility

## The First Studies of Low-Frequency and Rare Coding Variants

The first searches for low-frequency or rare variant T2D associations began even before the GWAS era. Following paradigms for Mendelian disease genetic mapping, many linkage and candidate gene studies were conducted for T2D in the late 1990s and early 2000s [34]. Other than an association near *TCF7L2* [35] (still the largest genetic contributor to T2D risk), these studies produced few replicable associations [34, 36, 37] and today are cited less so for their discoveries and more so as cautionary contrasts to the statistical rigor of GWAS [1, 13].

The first large-scale sequencing studies of T2D focused on genes with prior genetic links to T2D. One class of study focused on genes within GWAS regions, showing *MTNR1B* [38], *SLC30A8* [39, 40], *PPARG* [41], and *HNF1A* [42] to harbor collections of rare variants with moderate (OR 2–7) effects on T2D risk. Notably, in each case, stringent filtering of variants was necessary to reveal an association: the *SLC30A8* association was detected with the small fraction of variants predicted to truncate *SLC30A8*-encoded protein, while systematic characterizations of rare variants in (gene-specific) assays were needed to identify associations for *MTNR1B*, *PPARG*, and *HNF1A*. Furthermore, each of these genes was already widely believed (prior to the sequencing studies) to mediate the original GWAS association; early sequencing studies were less successful at identifying truly novel GWAS effector genes [40, 43].

A second class of targeted sequencing studies focused on genes for Maturity Onset Diabetes of the Young (MODY) or other Mendelian diseases with clinical similarities to T2D. Beginning with small studies that showed rare variants in MODY genes to have effects on T2D risk in the general population [44]—albeit with penetrances much lower than might have been expected—and continuing with larger studies that provided stronger statistical evidence of association [27, 44, 45••, 46, 47], MODY genes have been consistently shown to harbor not only rare variants that cause early onset Mendelian diseases but also a broader “allelic series” of variants that predispose to the later onset form of T2D. These findings are now widely interpreted as evidence that MODY and T2D are not distinct conditions but rather opposite extremes of a continuum of diabetes subtypes [15, 48, 49].

As exome sequencing, the exome array, and sequence-based imputation reference panels began to mature, the first genome-wide scans for rare and low-frequency variant T2D associations began to appear. The earliest exome array studies relevant to T2D were focused on glycemic traits; while some coding variants of moderate effect emerged from these studies (e.g., *PAM* for insulinogenic index [50], *G6PC2* for fasting glucose [51, 52], and *AKT2* for fasting insulin [53]), the number of significant associations was much smaller than would be expected from the hypotheses positing large contributions of rare variants to complex trait heritability. Early T2D sequencing studies [46, 47, 54, 55] (each in a few thousand individuals) similarly were successful at identifying some, but not many, novel rare or low-frequency coding variant T2D associations (e.g., in *PAM* [47], *PDX1* [47], *HNF1A* [46], and *ADCY3* [56]). Perhaps the biggest lesson to emerge from these investigations is the value offered by studies of populations either isolated or subject to historical bottlenecks (e.g., Iceland [47], Mexico [46], Finland [53], Greenland [56, 57]) as a means to identify strong-effect T2D variants that have, by chance (e.g. genetic drift) or perhaps even positive selection, risen to moderate (or even high) frequency.

Early studies of low-frequency and rare variation thus suggested that rare variant hypotheses might have been overly optimistic in their predictions about the contribution of rare variation to T2D. However, a definitive assessment of these hypotheses would require global and systematic analyses of larger datasets.

## An Emerging Picture of T2D Genetic Architecture

While a study of a few thousand sequenced individuals might be expected to detect a substantial number of rare variant associations under optimistic models, more measured early-stage simulations [58, 59] and analytical calculations [60] had predicted that tens of thousands of sequenced individuals would be required for reasonable power to interrogate the rare variant hypothesis for most complex diseases. An early sequencing study of 1000 T2D cases and 1000 controls, for example, had power to exclude only extreme models in which rare variants in < 20 genes explained the majority of T2D risk [55]. The lack of rare variant associations from early studies did not, therefore, rule out rare variant models for T2D, and a systematic simulation study showed that, prior to large-scale sequencing studies, rare and common variant models could each be constructed as consistent with empirical T2D genetic associations [61•].

The study that took the largest step toward constraining potential T2D genetic architectures was published in 2016 [45••], analyzing ~ 13,000 multi-ethnic exomes, ~ 2700 whole European ancestry genomes, ~ 80,000 samples genotyped on the exome array, and ~ 44,000 samples with genotypes imputed from a whole-genome sequence reference panel enriched for T2D cases. Using these data collectively, the study provided insights into all three major hypotheses about the role of rare and low-frequency variants in T2D genetic susceptibility. First, despite near-complete variant ascertainment in a modest-size European ancestry sample, only one low-frequency variant (a previously reported noncoding variant in *CCND2* [47]) achieved genome-wide significance, enabling quantitative bounds on the T2D effect sizes of low-frequency variants, which, in short, rejected models proposing a significant number of low-frequency strong-effect T2D variants. Second, simulated rare variant models predicted far more rare and low-frequency variant associations than were observed empirically, instead supporting a T2D genetic architecture characterized by many modest-effect common variants. Third, no rare variants could plausibly explain any significant T2D GWAS signals, rejecting synthetic associations as a common phenomenon for T2D. A fourth finding of the study was that no gene-level coding variant associations reached exome-wide significance, although the implications of this finding for the validity of rare variant models were not pursued in detail.

Since the publication of the 2016 study, four other large-scale studies have further constrained the contribution of rare and low-frequency variants to T2D susceptibility. The first study [62] performed deep whole-genome sequencing of 20 large Hispanic pedigrees (spanning ~ 1000 individuals), providing the opportunity to observe and analyze multiple copies of extremely rare variants (e.g., those private to a family). Although the power of the study design was validated by the identification of several rare variant associations with gene expression (cis-expression quantitative trait loci), no evidence of large-effect rare variant associations was observed for T2D in these families.

The second study [63••] applied the exome array to ~ 450,000 samples (~ 80,000 with T2D), significantly increasing power to interrogate MAF > 0.5% coding variation for T2D association. Although the study identified 40 coding variant associations, only five had observed MAF < 5% and none had observed OR > 1.4, strongly suggesting the fruitlessness of searches for low-frequency or common coding variants with even moderate effects on T2D risk. Furthermore, through fine mapping with densely imputed GWAS data, < 50% of the 40 coding variants identified in the study were shown to be causally linked to T2D risk, with the remainder likely proxies for nearby noncoding causal variants. Coding variant associations therefore cannot be immediately assumed to implicate specific variants or genes, although (because most of the 40 associations analyzed in the study were observed with common variants) the proportion of rare coding variant T2D associations that are causal may well be significantly higher.

The third study [64••] used HRC-based imputation to analyze ~ 900,000 European samples (~ 75,000 with T2D), providing even greater power to detect T2D associations with variants as rare as MAF~ 0.1% (although imputation quality, and therefore effective sample size, is lower for rarer variants). This study produced by far the largest catalog of low-frequency and rare variant associations to date for T2D, identifying associations with 56 low-frequency (0.5% < MAF < 5%) and 14 rare (MAF < 0.5%) variants across 60 loci; many of these variants are nearby but independent from common variants identified by earlier GWAS. Although variant OR estimates were not independently validated (and may be overestimates), some of the identified low-frequency variants had moderate to high estimated effects on T2D risk, with 14 having observed OR > 2 and two having observed OR ~ 8. However, only seven of the 56 low-frequency variant associations lie within coding regions, and all of these had estimated OR < 2. Collectively, low-frequency variant associations in the study were estimated to explain 15× less T2D heritability than were common variant associations in the same study (1.13% vs. 16.3%), implying the heritability explained by low-frequency coding variants to be even lower (by perhaps an order of magnitude).

These three studies progressively limited the role of low-frequency and rare coding variants in T2D susceptibility; however, they collectively ascertained at most only a small fraction of rare coding variation in the population. The final, and most recent, large-scale genetic study of T2D [65••] used exome sequencing in five major ethnic groups to analyze ~ 45,000 samples (~ 20,000 with T2D) across ~ 3M coding variants, ~ 95% of which are rare and ~ 90% of which are absent from exome array or HRC-based imputation studies. This study, as expected, identified essentially no novel coding single-variant associations (only one low-frequency variant in the known obesity and T2D gene *MC4R*). However, it did demonstrate, for the first time, exome-wide significant gene-level associations (*PAM*, *MC4R*, and *SLC30A8*). Notably, these three genes had been previously implicated in T2D via GWAS, and the rare variants contributing to the gene-level signals explain significantly less T2D heritability than do the nearby independently associated common variants.

Nonetheless, exome sequencing at this scale did reveal evidence for pervasive rare variant associations across T2D-relevant genes. Twelve gene sets, defined based on prior evidence from mouse models, T2D drug targets, or monogenic diabetes, each exhibited significantly more rare variant gene-level associations than expected by chance. Individually, the gene-level associations were modest at best, requiring (in the case of T2D drug targets) perhaps 500K–1M samples to be detected at exome-wide statistical significance. However, these results suggest that, once a gene is established as relevant to T2D, it might reasonably be expected to carry a rare coding variant allelic series that could be mined for more insight into its function.

**Fig. 1:**
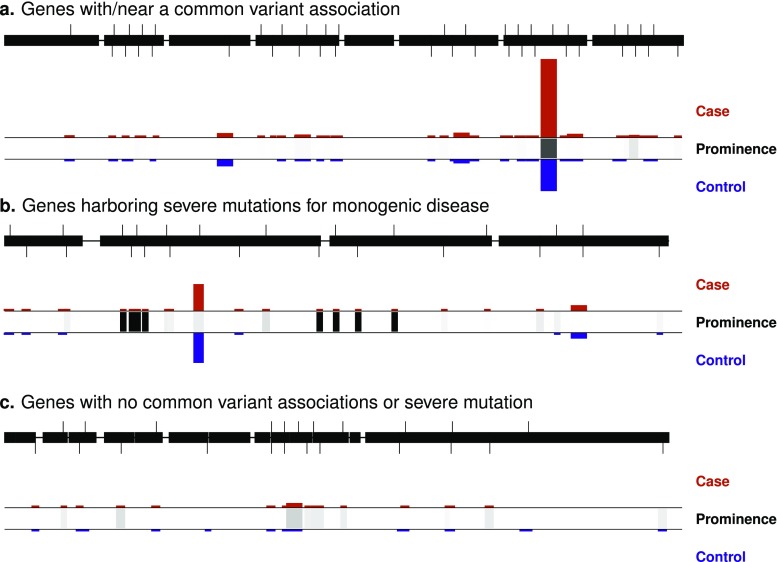
Profiles of rare coding variation in T2D-relevant genes. Based on recent empirical evidence, many T2D-relevant genes seem likely to harbor a series of rare coding variant associations. However, based on empirical aggregate effect sizes, typical rare variant associations may require an order of magnitude more exome sequences to detect than are available today. **a** Some genes, by chance, will lie near or contain a low-frequency or common variant T2D association, as is the case for the three exome-wide significant T2D gene-level associations identified to date. Such genes will likely be detected by GWAS or exome array single-variant analysis long before they are detected by exome sequence gene-level analysis. **b** Some genes will harbor extremely rare, severe mutations associated with a monogenic form of diabetes. Given evidence of a genetic overlap between monogenic forms of diabetes and T2D, these genes are strong candidates to harbor an allelic series of variants associated with T2D. **c** Many genes will only harbor a rare variant T2D gene-level association. Based on empirically observed aggregate effect sizes, these genes will likely be very hard to identify for the foreseeable future. For each example gene, variants are shown as tics on the transcript map; *red and blue bars* indicate variant case and control frequencies, respectively, and *black boxes* indicate the variant’s “prominence” (e.g., detectability via GWAS or a Mendelian gene mapping study)

In the last 3 years, a clearer picture of T2D genetic architecture has therefore begun to emerge. The set of rare and low-frequency coding variants with effect sizes large enough to be detected via single-variant analysis of even 1 million samples appears quite limited, even though ~ 350 independent common variants—most of them noncoding—have been identified from those same studies. This does not imply that rare variants play no role in T2D susceptibility; indeed, the most recent T2D exome sequencing study suggests that rare variant gene-level signals may be more of a norm than a surprise in T2D-relevant genes [65••], supporting other studies that have shown an excess of associations (genome-wide) in coding exons [45••, 66, 67]. However, rare variant signals have effect sizes significantly lower than might have been expected by optimistic early hypotheses and are often detectable only after the disease relevance of gene has been established (e.g., at a relaxed significance threshold justified by the gene’s “prior” evidence). For this reason, in the past and likely in the near future, most T2D rare variant signals have been or will be identified within genes harboring additional variants that, by chance, either (a) cause an extreme form of diabetes (e.g., MODY) or (b) have risen to sufficient frequency to be detectable via GWAS (Fig. 1).

## The Use of Coding Variants to Understand Disease Biology

Although it seems likely that rare and low-frequency variants contribute modestly to T2D heritability, they may still play a significant role in efforts to understand disease biology or design new therapies. It has long been appreciated that a variant need not explain much heritability to offer valuable disease insight [12, 13, 15], as a modest-effect variant could point to a gene of which larger therapeutic perturbations may have a significant impact [68] or to a pathway that might suggest an important new disease mechanism [69]. Translating genetic associations to biological function—and then investigating how natural or potential therapeutic alteration of these functions affects disease risk or progression—represents the most pressing current and future challenge in complex disease research [70].

Rare coding variants offer unique value in this endeavor. Because they localize to protein sequence, and because they are less likely to be highly correlated to hidden causal variants via linkage disequilibrium than are common variants, they can directly implicate genes in disease pathogenesis. Furthermore, since their effects are easier to interpret than those of noncoding variants, and because they can be introduced into model systems and evaluated for effects on a variety of molecular or cellular processes, they can help basic researchers to experimentally evaluate the downstream effects of a gene on a biological process. The empirical difficulty of identifying T2D rare variant associations limits their ability to identify novel disease loci genes. However, once a gene is hypothesized to be involved in disease, rare coding variants can provide valuable “handles” on the gene to probe the relationship between its function and disease (Table 2).

**Table 2:** The role of common and rare variation in future T2D genetic studies

Goal	Common variants	Rare coding variants
Locus discovery	GWAS in large sample sizes, imputed from progressively larger whole-genome sequence reference panels	Limited role for the foreseeable future (and possibly longer) due to significantly greater efficiency of GWAS
Reverse genetics	Limited role due to difficulties in identifying variants with clear molecular function	Identify individuals with loss of function mutations (“human knockouts” or haploinsufficiency), or severe missense mutations and analyze deep phenotypes
Biological function	Determine mechanism of original common variant association through functional genomic predictions, genome editing, and readouts from cellular/animal models	Characterize an “allelic series” of missense mutations to assess the molecular and cellular consequences of varied gene perturbations
Therapeutic translation	Of potential clinical utility to define subgroups or stratify populations through common variant polygenic risk scores	Use coding variants to link molecular and cellular readouts (effects of variants on an assay) to physiological phenotypes (genetic associations of the same variants) and potentially identify putative drug targets

Future studies to understand the biology of T2D and identify potential new therapies will require a combination of approaches to identify new genetic associations (Locus discovery), evaluate the role candidate genes play in human disease (Reverse genetics), translate associations to biological insights (Biological function), and suggest new therapies (Therapeutic translation). The table summarizes potential strategies to use common and rare variants toward each goal

The identification and subsequent characterization of *SLC30A8* provides an illustrative example of this approach. A coding variant in *SLC30A8* was one of the earliest findings of T2D GWAS [71], and the gene immediately piqued research and therapeutic interest: its protein product ZnT8 is expressed mainly in pancreatic islets and transports zinc into insulin-containing granules, thus contributing to insulin processing and storage. Because of its known function, the initial hypothesis was that reduced ZnT8 activity would increase risk of T2D [72, 73]; however, when numerous *Slc30a8* knockout mice were subsequently created and tested for a variety of glycemic phenotypes, no consistent effects on hyperglycemia emerged [73–77].

It was therefore surprising when 12 rare protein-truncating variants in *SLC30A8* were shown to associate, in aggregate, with *protection* from T2D [40]. While these data offered no insight into a potential protective mechanism, their clear predicted molecular effects (reduced ZnT8 activity) and expression within the full human system (rather than mice) prompted re-evaluation of the link between ZnT8 and T2D. After several years of further research, a recent mouse model of the most common *SLC30A8* rare protein-truncating variant offered a potential mechanism for T2D protection, through increased first-phase insulin secretion [78•].

Much still remains to be determined about the mechanism linking ZnT8 loss of function to increased insulin secretion, not only in animal models but also in humans. One limitation of using the 12 *SLC30A8* protein-truncating variants to explore this relationship is that all are expected to result in human haploinsufficiency, and they thus do not inform on potential effects of either full loss of function or more measured reductions in ZnT8 activity. Coding variants could help address this knowledge gap in two respects. First, if *SLC30A8* “human knockouts”—or individuals with homozygous or compound heterozygous loss of function mutations—were identified [18, 79], they could be deeply characterized for various phenotypes to better understand the intermediate human physiological processes responsible for T2D protection. Second, the exome-wide significant series of > 100 *SLC30A8* protective missense alleles from the most recent T2D exome sequencing study [65••] could be used to probe the *SLC30A8* “dose-response” curve: each allele could be introduced into a molecular or cellular assay (such as zinc transport or insulin secretion), and their effects on these assays could then be compared to their T2D protective effects to calibrate the relationship between T2D risk and the biological process measured by the assay. Particularly interesting variants could be subsequently introduced into an *Slc30a8* mouse model for further characterization. This paradigm has been previously demonstrated for T2D in the context of *PPARG* and insulin resistance, in which simultaneous effects of rare coding variants on T2D risk (when analyzed in a genetic study) and adipogenesis (when introduced into a cellular assay) validated the gene’s mechanism of action [41]. Furthermore, once an assay has been established as a proxy for human disease risk, it represents an attractive asset for future therapeutic screens [3].

## Conclusion

The past 5 years of T2D genetic research have addressed the goal of understanding T2D genetic architecture: it is now highly likely that T2D genetic risk is mostly determined by many common, modest-effect regulatory variants rather than a few rare, large-effect coding variants. The jury is still out on the extent to which rare and low-frequency variants of individually large effect might aid disease risk prediction in selected groups: only a few examples of low-frequency variants with large effects on T2D risk have been identified [57, 80], and these are typically highly population-specific (and in fact often common in the particular population). Collections of large-effect rare variants may reach sufficient aggregate frequency for useful risk prediction in some genes, but to provide clinical utility, these variants will likely require functional characterization [42, 81•, 82, 83], a significant limitation for variants in novel genes. By contrast, polygenic risk scores constructed from many common variants have steadily increased in predictive power: those constructed from the latest T2D GWAS show a ninefold difference in disease prevalence for individuals at the high and low extremes of risk [64••].

The most effective path toward the goal of T2D biological discovery seems likely to include both traditional GWAS approaches as well as novel methods incorporating rare coding variant analyses (Table 2). GWAS approaches—which will only increase in power to interrogate low-frequency and rare variants as imputation reference panels expand—will likely persist for the foreseeable future as the most efficient means to identify genomic loci associated with disease. Coding variants seem better placed to help understand the functional consequences of an association by providing a variety of gene perturbations that can be both experimentally and phenotypically characterized, an area in which improved methods for conducting and dissecting gene-level tests could have a significant impact. Additionally, it seems plausible that some (maybe many) disease-relevant genes may prove undetectable by GWAS, as an association depends not only on the disease relevance of a gene but also on the historical emergence of a (reasonably common) genetic variant that sufficiently perturbs its function [17]. For T2D-relevant genes lacking a GWAS association—which might be identified as therapeutic or biological candidates from high-throughput functional screens or because they participate in a hypothesized disease-relevant pathway—coding variants offer the opportunity to conduct “reverse genetic” analyses in which variants that alter function of the gene are identified and then characterized for their effects on human phenotypes.

Although rare and low-frequency coding variants are not the panacea that some optimistic genetic models proposed after the first T2D GWAS, they are not as irrelevant to T2D as might be naively inferred from the now-appreciated dominant contribution of common noncoding variants to disease heritability. Instead, they provide one of several genetic tools necessary to understand and ultimately develop better treatments for complex diseases such as T2D—in particular, a tool for probing and refining gene function. The true revolution enabled by rare coding variation may therefore be not the previous phase of complex disease discovery but the next phase of complex disease association functional and clinical translation.

## References

[1] Altshuler D, Daly MJ, Lander ES (2008). Genetic mapping in human disease. Science..

[2] Manolio TA, Collins FS, Cox NJ, Goldstein DB, Hindorff LA, Hunter DJ, McCarthy MI, Ramos EM, Cardon LR, Chakravarti A, Cho JH, Guttmacher AE, Kong A, Kruglyak L, Mardis E, Rotimi CN, Slatkin M, Valle D, Whittemore AS, Boehnke M, Clark AG, Eichler EE, Gibson G, Haines JL, Mackay TFC, McCarroll SA, Visscher PM (2009). Finding the missing heritability of complex diseases. Nature..

[3] Plenge RM, Scolnick EM, Altshuler D (2013). Validating therapeutic targets through human genetics. Nat Rev Drug Discov.

[4] Botstein D, Risch N (2003). Discovering genotypes underlying human phenotypes: past successes for mendelian disease, future approaches for complex disease. Nat Genet.

[5] Cirulli ET, Goldstein DB (2010). Uncovering the roles of rare variants in common disease through whole-genome sequencing. Nat Rev Genet.

[6] Cooper GM, Shendure J (2011). Needles in stacks of needles: finding disease-causal variants in a wealth of genomic data. Nat Rev Genet.

[7] McClellan J, King MC (2010). Genetic heterogeneity in human disease. Cell..

[8] Cohen J, Pertsemlidis A, Kotowski IK, Graham R, Garcia CK, Hobbs HH (2005). Low LDL cholesterol in individuals of African descent resulting from frequent nonsense mutations in PCSK9. Nat Genet.

[9] Cohen JC, Boerwinkle E, Mosley TH, Hobbs HH (2006). Sequence variations in PCSK9, low LDL, and protection against coronary heart disease. N Engl J Med.

[10] Stein EA, Mellis S, Yancopoulos GD, Stahl N, Logan D, Smith WB, Lisbon E, Gutierrez M, Webb C, Wu R, du Y, Kranz T, Gasparino E, Swergold GD (2012). Effect of a monoclonal antibody to PCSK9 on LDL cholesterol. N Engl J Med.

[11] Hindorff LA, Sethupathy P, Junkins HA, Ramos EM, Mehta JP, Collins FS, Manolio TA (2009). Potential etiologic and functional implications of genome-wide association loci for human diseases and traits. Proc Natl Acad Sci U S A.

[12] Hirschhorn JN (2009). Genomewide association studies--illuminating biologic pathways. N Engl J Med.

[13] Goldstein DB (2009). Common genetic variation and human traits. N Engl J Med.

[14] Bodmer W, Bonilla C (2008). Common and rare variants in multifactorial susceptibility to common diseases. Nat Genet.

[15] Lupski JR, Belmont JW, Boerwinkle E, Gibbs RA (2011). Clan genomics and the complex architecture of human disease. Cell..

[16] Kryukov GV, Pennacchio LA, Sunyaev SR (2007). Most rare missense alleles are deleterious in humans: implications for complex disease and association studies. Am J Hum Genet.

[17] Pritchard JK (2001). Are rare variants responsible for susceptibility to complex diseases?. Am J Hum Genet.

[18] Saleheen D, Natarajan P, Armean IM, Zhao W, Rasheed A, Khetarpal SA, Won HH, Karczewski KJ, O’Donnell-Luria AH, Samocha KE, Weisburd B, Gupta N, Zaidi M, Samuel M, Imran A, Abbas S, Majeed F, Ishaq M, Akhtar S, Trindade K, Mucksavage M, Qamar N, Zaman KS, Yaqoob Z, Saghir T, Rizvi SNH, Memon A, Hayyat Mallick N, Ishaq M, Rasheed SZ, Memon FUR, Mahmood K, Ahmed N, Do R, Krauss RM, MacArthur DG, Gabriel S, Lander ES, Daly MJ, Frossard P, Danesh J, Rader DJ, Kathiresan S (2017). Human knockouts and phenotypic analysis in a cohort with a high rate of consanguinity. Nature..

[19] Tennessen JA, Bigham AW, O'Connor TD, Fu W, Kenny EE, Gravel S, McGee S, Do R, Liu X, Jun G, Kang HM, Jordan D, Leal SM, Gabriel S, Rieder MJ, Abecasis G, Altshuler D, Nickerson DA, Boerwinkle E, Sunyaev S, Bustamante CD, Bamshad MJ, Akey JM, Broad GO, Seattle GO, on behalf of the NHLBI Exome Sequencing Project (2012). Evolution and functional impact of rare coding variation from deep sequencing of human exomes. Science..

[20] Keinan A, Clark AG (2012). Recent explosive human population growth has resulted in an excess of rare genetic variants. Science..

[21] Dickson SP, Wang K, Krantz I, Hakonarson H, Goldstein DB (2010). Rare variants create synthetic genome-wide associations. PLoS Biol.

[22] Metzker ML (2010). Sequencing technologies - the next generation. Nat Rev Genet.

[23] Nejentsev S, Walker N, Riches D, Egholm M, Todd JA (2009). Rare variants of IFIH1, a gene implicated in antiviral responses, protect against type 1 diabetes. Science..

[24] Rivas MA, Beaudoin M, Gardet A, Stevens C, Sharma Y, Zhang CK (2011). Deep resequencing of GWAS loci identifies independent rare variants associated with inflammatory bowel disease. Nat Genet.

[25] Kiezun A, Garimella K, Do R, Stitziel NO, Neale BM, McLaren PJ (2012). Exome sequencing and the genetic basis of complex traits. Nat Genet.

[26] Ng SB, Turner EH, Robertson PD, Flygare SD, Bigham AW, Lee C, Shaffer T, Wong M, Bhattacharjee A, Eichler EE, Bamshad M, Nickerson DA, Shendure J (2009). Targeted capture and massively parallel sequencing of 12 human exomes. Nature..

[27] Bansal V, Gassenhuber J, Phillips T, Oliveira G, Harbaugh R, Villarasa N, Topol EJ, Seufferlein T, Boehm BO (2017). Spectrum of mutations in monogenic diabetes genes identified from high-throughput DNA sequencing of 6888 individuals. BMC Med.

[28] McCarthy S, Das S, Kretzschmar W, Delaneau O, Wood AR, Teumer A, Kang HM, Fuchsberger C, Danecek P, Sharp K, Luo Y, Sidore C, Kwong A, Timpson N, Koskinen S, Vrieze S, Scott LJ, Zhang H, Mahajan A, Veldink J, Peters U, Pato C, van Duijn C, Gillies CE, Gandin I, Mezzavilla M, Gilly A, Cocca M, Traglia M, Angius A, Barrett JC, Boomsma D, Branham K, Breen G, Brummett CM, Busonero F, Campbell H, Chan A, Chen S, Chew E, Collins FS, Corbin LJ, Smith GD, Dedoussis G, Dorr M, Farmaki AE, Ferrucci L, Forer L, Fraser RM, Gabriel S, Levy S, Groop L, Harrison T, Hattersley A, Holmen OL, Hveem K, Kretzler M, Lee JC, McGue M, Meitinger T, Melzer D, Min JL, Mohlke KL, Vincent JB, Nauck M, Nickerson D, Palotie A, Pato M, Pirastu N, McInnis M, Richards JB, Sala C, Salomaa V, Schlessinger D, Schoenherr S, Slagboom PE, Small K, Spector T, Stambolian D, Tuke M, Tuomilehto J, van den Berg L, van Rheenen W, Volker U, Wijmenga C, Toniolo D, Zeggini E, Gasparini P, Sampson MG, Wilson JF, Frayling T, de Bakker PI, Swertz MA, McCarroll S, Kooperberg C, Dekker A, Altshuler D, Willer C, Iacono W, Ripatti S, Soranzo N, Walter K, Swaroop A, Cucca F, Anderson CA, Myers RM, Boehnke M, McCarthy M, Durbin R, Haplotype Reference Consortium (2016). A reference panel of 64,976 haplotypes for genotype imputation. Nat Genet.

[29] Lee S, Abecasis GR, Boehnke M, Lin X (2014). Rare-variant association analysis: study designs and statistical tests. Am J Hum Genet.

[30] Adzhubei Ivan, Jordan Daniel M., Sunyaev Shamil R. (2013). Predicting Functional Effect of Human Missense Mutations Using PolyPhen-2. Current Protocols in Human Genetics.

[31] Liu X, Wu C, Li C, Boerwinkle E (2016). dbNSFP v3.0: a one-stop database of functional predictions and annotations for human nonsynonymous and splice-site SNVs. Hum Mutat.

[32] Wu MC, Lee S, Cai T, Li Y, Boehnke M, Lin X (2011). Rare-variant association testing for sequencing data with the sequence kernel association test. Am J Hum Genet.

[33] Lee S, Emond MJ, Bamshad MJ, Barnes KC, Rieder MJ, Nickerson DA, Christiani DC, Wurfel MM, Lin X, NHLBI GO Exome Sequencing Project—ESP Lung Project Team (2012). Optimal unified approach for rare-variant association testing with application to small-sample case-control whole-exome sequencing studies. Am J Hum Genet.

[34] Bonnefond A, Froguel P (2015). Rare and common genetic events in type 2 diabetes: what should biologists know?. Cell Metab.

[35] Grant SF, Thorleifsson G, Reynisdottir I, Benediktsson R, Manolescu A, Sainz J (2006). Variant of transcription factor 7-like 2 (TCF7L2) gene confers risk of type 2 diabetes. Nat Genet.

[36] Guan W, Pluzhnikov A, Cox NJ, Boehnke M (2008). International Type 2 Diabetes Linkage Analysis C. Meta-analysis of 23 type 2 diabetes linkage studies from the International Type 2 Diabetes Linkage Analysis Consortium. Hum Hered.

[37] Hirschhorn JN, Lohmueller K, Byrne E, Hirschhorn K (2002). A comprehensive review of genetic association studies. Genet Med : Off J Am Coll Med Genet.

[38] Bonnefond A, Clement N, Fawcett K, Yengo L, Vaillant E, Guillaume JL (2012). Rare MTNR1B variants impairing melatonin receptor 1B function contribute to type 2 diabetes. Nat Genet.

[39] Billings LK, Jablonski KA, Ackerman RJ, Taylor A, Fanelli RR, McAteer JB, Guiducci C, Delahanty LM, Dabelea D, Kahn SE, Franks PW, Hanson RL, Maruthur NM, Shuldiner AR, Mayer-Davis EJ, Knowler WC, Florez JC, Diabetes Prevention Program Research Group, Rockville (2014). The influence of rare genetic variation in SLC30A8 on diabetes incidence and beta-cell function. J Clin Endocrinol Metab.

[40] Flannick J, Thorleifsson G, Beer NL, Jacobs SB, Grarup N, Burtt NP (2014). Loss-of-function mutations in SLC30A8 protect against type 2 diabetes. Nat Genet.

[41] Majithia AR, Flannick J, Shahinian P, Guo M, Bray MA, Fontanillas P, Gabriel SB, Rosen ED, Altshuler D, Flannick J, Manning AK, Hartl C, Agarwala V, Fontanillas P, Green T, Banks E, DePristo M, Poplin R, Shakir K, Fennell T, Njolstad PR, Altshuler D, Burtt N, Gabriel S, Fuchsberger C, Kang HM, Sim X, Ma C, Locke A, Blackwell T, Jackson A, Teslovich TM, Stringham H, Chines P, Kwan P, Huyghe J, Tan A, Jun G, Stitzel M, Bergman RN, Bonnycastle L, Tuomilehto J, Collins FS, Scott L, Mohlke K, Abecasis G, Boehnke M, Strom T, Gieger C, Nurasyid MM, Grallert H, Kriebel J, Ried J, Hrabe de Angelis M, Huth C, Meisinger C, Peters A, Rathmann W, Strauch K, Meitinger T, Kravic J, Algren P, Ladenvall C, Toumi T, Isomaa B, Groop L, Gaulton K, Moutsianas L, Rivas M, Pearson R, Mahajan A, Prokopenko I, Kumar A, Perry J, Howie B, van de Bunt M, Small K, Lindgren C, Lunter G, Robertson N, Rayner W, Morris A, Buck D, Hattersley A, Spector T, McVean G, Frayling T, Donnelly P, McCarthy M, Gupta N, Taylor H, Fox E, Cheh CN, Wilson JG, O'Donnell CJ, Kathiresan S, Hirschhorn J, Seidman JG, Gabriel S, Seidman C, Altshuler D, Williams AL, Jacobs SBR, Macias HM, Chagoya AH, Churchhouse C, Luna CM, Ortiz HG, Vazquez MJG, Burtt NP, Estrada K, Mercader JM, Ripke S, Manning AK, Neale B, Stram DO, Lopez JCF, Hidalgo SR, Delfin IA, Hernandez AM, Cruz FC, Caamal EM, Monsalve CR, Andrade SI, Cordova E, Arellano ER, Soberon X, Villalpando MEG, Monroe K, Wilkens L, Kolonel LN, le Marchand L, Riba L, Sanchez MLO, Guillen RR, Bautista IC, Torres MR, Hernandez LLM, Saenz T, Gomez D, Alvirde U, Onofrio RC, Brodeur WM, Gage D, Murphy J, Franklin J, Mahan S, Ardlie K, Crenshaw AT, Winckler W, Fennell T, MacArthur DG, Altshuler D, Florez JC, Haiman CA, Henderson BE, Salinas CAA, Villalpando CG, Orozco L, Luna TT, Abecasis G, Almeida M, Altshuler D, Asimit JL, Atzmon G, Barber M, Beer NL, Bell GI, Below J, Blackwell T, Blangero J, Boehnke M, Bowden DW, Burtt N, Chambers J, Chen H, Chen P, Chines PS, Choi S, Churchhouse C, Cingolani P, Cornes BK, Cox N, Williams AGD, Duggirala R, Dupuis J, Dyer T, Feng S, Tajes JF, Ferreira T, Fingerlin TE, Flannick J, Florez J, Fontanillas P, Frayling TM, Fuchsberger C, Gamazon ER, Gaulton K, Ghosh S, Gloyn A, Grossman RL, Grundstad J, Hanis C, Heath A, Highland H, Hirokoshi M, Huh IS, Huyghe JR, Ikram K, Jablonski KA, Kim YJ, Jun G, Kato N, Kim J, King CR, Kooner J, Kwon MS, Im HK, Laakso M, Lam KKY, Lee J, Lee S, Lee S, Lehman DM, Li H, Lindgren CM, Liu X, Livne OE, Locke AE, Mahajan A, Maller JB, Manning AK, Maxwell TJ, Mazoure A, McCarthy MI, Meigs JB, Min B, Mohlke KL, Morris A, Musani S, Nagai Y, Ng MCY, Nicolae D, Oh S, Palmer N, Park T, Pollin TI, Prokopenko I, Reich D, Rivas MA, Scott LJ, Seielstad M, Cho YS, Tai ES, Sim X, Sladek R, Smith P, Tachmazidou I, Teslovich TM, Torres J, Trubetskoy V, Willems SM, Williams AL, Wilson JG, Wiltshire S, Won S, Wood AR, Xu W, Teo YY, Yoon J, Lee JY, Zawistowski M, Zeggini E, Zhang W, Zollner S, GoT2D Consortium, NHGRI JHS/FHS Allelic Spectrum Project, SIGMA T2D Consortium, T2D-GENES Consortium (2014). Rare variants in PPARG with decreased activity in adipocyte differentiation are associated with increased risk of type 2 diabetes. Proc Natl Acad Sci U S A.

[42] Najmi LA, Aukrust I, Flannick J, Molnes J, Burtt N, Molven A, Groop L, Altshuler D, Johansson S, Bjørkhaug L, Njølstad PR (2017). Functional investigations of HNF1A identify rare variants as risk factors for type 2 diabetes in the general population. Diabetes..

[43] Maller JB, Mcvean G, Byrnes J, Vukcevic D, Palin K, Wellcome Trust Case Control Consortium (2012). Bayesian refinement of association signals for 14 loci in 3 common diseases. Nat Genet.

[44] Flannick J, Beer NL, Bick AG, Agarwala V, Molnes J, Gupta N, Burtt NP, Florez JC, Meigs JB, Taylor H, Lyssenko V, Irgens H, Fox E, Burslem F, Johansson S, Brosnan MJ, Trimmer JK, Newton-Cheh C, Tuomi T, Molven A, Wilson JG, O'Donnell CJ, Kathiresan S, Hirschhorn JN, Njølstad PR, Rolph T, Seidman JG, Gabriel S, Cox DR, Seidman CE, Groop L, Altshuler D (2013). Assessing the phenotypic effects in the general population of rare variants in genes for a dominant Mendelian form of diabetes. Nat Genet.

[45.••] Fuchsberger C, Flannick J, Teslovich TM, Mahajan A, Agarwala V, Gaulton KJ, Ma C, Fontanillas P, Moutsianas L, McCarthy DJ, Rivas MA, Perry JRB, Sim X, Blackwell TW, Robertson NR, Rayner NW, Cingolani P, Locke AE, Tajes JF, Highland HM, Dupuis J, Chines PS, Lindgren CM, Hartl C, Jackson AU, Chen H, Huyghe JR, van de Bunt M, Pearson RD, Kumar A, Müller-Nurasyid M, Grarup N, Stringham HM, Gamazon ER, Lee J, Chen Y, Scott RA, Below JE, Chen P, Huang J, Go MJ, Stitzel ML, Pasko D, Parker SCJ, Varga TV, Green T, Beer NL, Day-Williams AG, Ferreira T, Fingerlin T, Horikoshi M, Hu C, Huh I, Ikram MK, Kim BJ, Kim Y, Kim YJ, Kwon MS, Lee J, Lee S, Lin KH, Maxwell TJ, Nagai Y, Wang X, Welch RP, Yoon J, Zhang W, Barzilai N, Voight BF, Han BG, Jenkinson CP, Kuulasmaa T, Kuusisto J, Manning A, Ng MCY, Palmer ND, Balkau B, Stančáková A, Abboud HE, Boeing H, Giedraitis V, Prabhakaran D, Gottesman O, Scott J, Carey J, Kwan P, Grant G, Smith JD, Neale BM, Purcell S, Butterworth AS, Howson JMM, Lee HM, Lu Y, Kwak SH, Zhao W, Danesh J, Lam VKL, Park KS, Saleheen D, So WY, Tam CHT, Afzal U, Aguilar D, Arya R, Aung T, Chan E, Navarro C, Cheng CY, Palli D, Correa A, Curran JE, Rybin D, Farook VS, Fowler SP, Freedman BI, Griswold M, Hale DE, Hicks PJ, Khor CC, Kumar S, Lehne B, Thuillier D, Lim WY, Liu J, van der Schouw YT, Loh M, Musani SK, Puppala S, Scott WR, Yengo L, Tan ST, Taylor HA, Thameem F, Wilson G, Wong TY, Njølstad PR, Levy JC, Mangino M, Bonnycastle LL, Schwarzmayr T, Fadista J, Surdulescu GL, Herder C, Groves CJ, Wieland T, Bork-Jensen J, Brandslund I, Christensen C, Koistinen HA, Doney ASF, Kinnunen L, Esko T, Farmer AJ, Hakaste L, Hodgkiss D, Kravic J, Lyssenko V, Hollensted M, Jørgensen ME, Jørgensen T, Ladenvall C, Justesen JM, Käräjämäki A, Kriebel J, Rathmann W, Lannfelt L, Lauritzen T, Narisu N, Linneberg A, Melander O, Milani L, Neville M, Orho-Melander M, Qi L, Qi Q, Roden M, Rolandsson O, Swift A, Rosengren AH, Stirrups K, Wood AR, Mihailov E, Blancher C, Carneiro MO, Maguire J, Poplin R, Shakir K, Fennell T, DePristo M, Hrabé de Angelis M, Deloukas P, Gjesing AP, Jun G, Nilsson P, Murphy J, Onofrio R, Thorand B, Hansen T, Meisinger C, Hu FB, Isomaa B, Karpe F, Liang L, Peters A, Huth C, O’Rahilly SP, Palmer CNA, Pedersen O, Rauramaa R, Tuomilehto J, Salomaa V, Watanabe RM, Syvänen AC, Bergman RN, Bharadwaj D, Bottinger EP, Cho YS, Chandak GR, Chan JCN, Chia KS, Daly MJ, Ebrahim SB, Langenberg C, Elliott P, Jablonski KA, Lehman DM, Jia W, Ma RCW, Pollin TI, Sandhu M, Tandon N, Froguel P, Barroso I, Teo YY, Zeggini E, Loos RJF, Small KS, Ried JS, DeFronzo RA, Grallert H, Glaser B, Metspalu A, Wareham NJ, Walker M, Banks E, Gieger C, Ingelsson E, Im HK, Illig T, Franks PW, Buck G, Trakalo J, Buck D, Prokopenko I, Mägi R, Lind L, Farjoun Y, Owen KR, Gloyn AL, Strauch K, Tuomi T, Kooner JS, Lee JY, Park T, Donnelly P, Morris AD, Hattersley AT, Bowden DW, Collins FS, Atzmon G, Chambers JC, Spector TD, Laakso M, Strom TM, Bell GI, Blangero J, Duggirala R, Tai ES, McVean G, Hanis CL, Wilson JG, Seielstad M, Frayling TM, Meigs JB, Cox NJ, Sladek R, Lander ES, Gabriel S, Burtt NP, Mohlke KL, Meitinger T, Groop L, Abecasis G, Florez JC, Scott LJ, Morris AP, Kang HM, Boehnke M, Altshuler D, McCarthy MI (2016). The genetic architecture of type 2 diabetes. Nature..

[46] Estrada K, Aukrust I, Bjorkhaug L, Burtt NP, Mercader JM, SIGMA Type 2 Diabetes Consortium (2014). Association of a low-frequency variant in HNF1A with type 2 diabetes in a Latino population. Jama..

[47] Steinthorsdottir V, Thorleifsson G, Sulem P, Helgason H, Grarup N, Sigurdsson A, Helgadottir HT, Johannsdottir H, Magnusson OT, Gudjonsson SA, Justesen JM, Harder MN, Jørgensen ME, Christensen C, Brandslund I, Sandbæk A, Lauritzen T, Vestergaard H, Linneberg A, Jørgensen T, Hansen T, Daneshpour MS, Fallah MS, Hreidarsson AB, Sigurdsson G, Azizi F, Benediktsson R, Masson G, Helgason A, Kong A, Gudbjartsson DF, Pedersen O, Thorsteinsdottir U, Stefansson K (2014). Identification of low-frequency and rare sequence variants associated with elevated or reduced risk of type 2 diabetes. Nat Genet.

[48] Flannick J, Johansson S, Njolstad PR (2016). Common and rare forms of diabetes mellitus: towards a continuum of diabetes subtypes. Nat Rev Endocrinol.

[49] Katsanis N (2016). The continuum of causality in human genetic disorders. Genome Biol.

[50] Huyghe JR, Jackson AU, Fogarty MP, Buchkovich ML, Stancakova A, Stringham HM (2013). Exome array analysis identifies new loci and low-frequency variants influencing insulin processing and secretion. Nat Genet.

[51] Mahajan A, Sim X, Ng HJ, Manning A, Rivas MA, Highland HM, Locke AE, Grarup N, Im HK, Cingolani P, Flannick J, Fontanillas P, Fuchsberger C, Gaulton KJ, Teslovich TM, Rayner NW, Robertson NR, Beer NL, Rundle JK, Bork-Jensen J, Ladenvall C, Blancher C, Buck D, Buck G, Burtt NP, Gabriel S, Gjesing AP, Groves CJ, Hollensted M, Huyghe JR, Jackson AU, Jun G, Justesen JM, Mangino M, Murphy J, Neville M, Onofrio R, Small KS, Stringham HM, Syvänen AC, Trakalo J, Abecasis G, Bell GI, Blangero J, Cox NJ, Duggirala R, Hanis CL, Seielstad M, Wilson JG, Christensen C, Brandslund I, Rauramaa R, Surdulescu GL, Doney ASF, Lannfelt L, Linneberg A, Isomaa B, Tuomi T, Jørgensen ME, Jørgensen T, Kuusisto J, Uusitupa M, Salomaa V, Spector TD, Morris AD, Palmer CNA, Collins FS, Mohlke KL, Bergman RN, Ingelsson E, Lind L, Tuomilehto J, Hansen T, Watanabe RM, Prokopenko I, Dupuis J, Karpe F, Groop L, Laakso M, Pedersen O, Florez JC, Morris AP, Altshuler D, Meigs JB, Boehnke M, McCarthy MI, Lindgren CM, Gloyn AL, On Behalf of the T2D-GENES consortium and GoT2D consortium (2015). Identification and functional characterization of G6PC2 coding variants influencing glycemic traits define an effector transcript at the G6PC2-ABCB11 locus. PLoS Genet.

[52] Wessel J, Chu AY, Willems SM, Wang S, Yaghootkar H, Brody JA (2015). Low-frequency and rare exome chip variants associate with fasting glucose and type 2 diabetes susceptibility. Nat Commun.

[53] Manning A, Highland HM, Gasser J, Sim X, Tukiainen T, Fontanillas P, Grarup N, Rivas MA, Mahajan A, Locke AE, Cingolani P, Pers TH, Viñuela A, Brown AA, Wu Y, Flannick J, Fuchsberger C, Gamazon ER, Gaulton KJ, Im HK, Teslovich TM, Blackwell TW, Bork-Jensen J, Burtt NP, Chen Y, Green T, Hartl C, Kang HM, Kumar A, Ladenvall C, Ma C, Moutsianas L, Pearson RD, Perry JRB, Rayner NW, Robertson NR, Scott LJ, van de Bunt M, Eriksson JG, Jula A, Koskinen S, Lehtimäki T, Palotie A, Raitakari OT, Jacobs SBR, Wessel J, Chu AY, Scott RA, Goodarzi MO, Blancher C, Buck G, Buck D, Chines PS, Gabriel S, Gjesing AP, Groves CJ, Hollensted M, Huyghe JR, Jackson AU, Jun G, Justesen JM, Mangino M, Murphy J, Neville M, Onofrio R, Small KS, Stringham HM, Trakalo J, Banks E, Carey J, Carneiro MO, DePristo M, Farjoun Y, Fennell T, Goldstein JI, Grant G, Hrabé de Angelis M, Maguire J, Neale BM, Poplin R, Purcell S, Schwarzmayr T, Shakir K, Smith JD, Strom TM, Wieland T, Lindstrom J, Brandslund I, Christensen C, Surdulescu GL, Lakka TA, Doney ASF, Nilsson P, Wareham NJ, Langenberg C, Varga TV, Franks PW, Rolandsson O, Rosengren AH, Farook VS, Thameem F, Puppala S, Kumar S, Lehman DM, Jenkinson CP, Curran JE, Hale DE, Fowler SP, Arya R, DeFronzo RA, Abboud HE, Syvänen AC, Hicks PJ, Palmer ND, Ng MCY, Bowden DW, Freedman BI, Esko T, Mägi R, Milani L, Mihailov E, Metspalu A, Narisu N, Kinnunen L, Bonnycastle LL, Swift A, Pasko D, Wood AR, Fadista J, Pollin TI, Barzilai N, Atzmon G, Glaser B, Thorand B, Strauch K, Peters A, Roden M, Müller-Nurasyid M, Liang L, Kriebel J, Illig T, Grallert H, Gieger C, Meisinger C, Lannfelt L, Musani SK, Griswold M, Taylor HA, Wilson G, Correa A, Oksa H, Scott WR, Afzal U, Tan ST, Loh M, Chambers JC, Sehmi J, Kooner JS, Lehne B, Cho YS, Lee JY, Han BG, Käräjämäki A, Qi Q, Qi L, Huang J, Hu FB, Melander O, Orho-Melander M, Below JE, Aguilar D, Wong TY, Liu J, Khor CC, Chia KS, Lim WY, Cheng CY, Chan E, Tai ES, Aung T, Linneberg A, Isomaa B, Meitinger T, Tuomi T, Hakaste L, Kravic J, Jørgensen ME, Lauritzen T, Deloukas P, Stirrups KE, Owen KR, Farmer AJ, Frayling TM, O'Rahilly SP, Walker M, Levy JC, Hodgkiss D, Hattersley AT, Kuulasmaa T, Stančáková A, Barroso I, Bharadwaj D, Chan J, Chandak GR, Daly MJ, Donnelly PJ, Ebrahim SB, Elliott P, Fingerlin T, Froguel P, Hu C, Jia W, Ma RCW, McVean G, Park T, Prabhakaran D, Sandhu M, Scott J, Sladek R, Tandon N, Teo YY, Zeggini E, Watanabe RM, Koistinen HA, Kesaniemi YA, Uusitupa M, Spector TD, Salomaa V, Rauramaa R, Palmer CNA, Prokopenko I, Morris AD, Bergman RN, Collins FS, Lind L, Ingelsson E, Tuomilehto J, Karpe F, Groop L, Jørgensen T, Hansen T, Pedersen O, Kuusisto J, Abecasis G, Bell GI, Blangero J, Cox NJ, Duggirala R, Seielstad M, Wilson JG, Dupuis J, Ripatti S, Hanis CL, Florez JC, Mohlke KL, Meigs JB, Laakso M, Morris AP, Boehnke M, Altshuler D, McCarthy MI, Gloyn AL, Lindgren CM (2017). A low-frequency inactivating AKT2 variant enriched in the finnish population is associated with fasting insulin levels and type 2 diabetes risk. Diabetes..

[54] Albrechtsen A, Grarup N, Li Y, Sparso T, Tian G, Cao H (2013). Exome sequencing-driven discovery of coding polymorphisms associated with common metabolic phenotypes. Diabetologia.

[55] Lohmueller KE, Sparso T, Li Q, Andersson E, Korneliussen T, Albrechtsen A (2013). Whole-exome sequencing of 2,000 Danish individuals and the role of rare coding variants in type 2 diabetes. Am J Hum Genet.

[56] Grarup N, Moltke I, Andersen MK, Dalby M, Vitting-Seerup K, Kern T, Mahendran Y, Jørsboe E, Larsen CVL, Dahl-Petersen IK, Gilly A, Suveges D, Dedoussis G, Zeggini E, Pedersen O, Andersson R, Bjerregaard P, Jørgensen ME, Albrechtsen A, Hansen T (2018). Loss-of-function variants in ADCY3 increase risk of obesity and type 2 diabetes. Nat Genet.

[57] Moltke I, Grarup N, Jorgensen ME, Bjerregaard P, Treebak JT, Fumagalli M (2014). A common Greenlandic TBC1D4 variant confers muscle insulin resistance and type 2 diabetes. Nature..

[58] Kryukov GV, Shpunt A, Stamatoyannopoulos JA, Sunyaev SR (2009). Power of deep, all-exon resequencing for discovery of human trait genes. Proc Natl Acad Sci U S A.

[59] Moutsianas L, Agarwala V, Fuchsberger C, Flannick J, Rivas MA, Gaulton KJ, Albers PK, McVean G, Boehnke M, Altshuler D, McCarthy MI, GoT2D Consortium (2015). The power of gene-based rare variant methods to detect disease-associated variation and test hypotheses about complex disease. PLoS Genet.

[60] Zuk O, Schaffner SF, Samocha K, Do R, Hechter E, Kathiresan S, Daly MJ, Neale BM, Sunyaev SR, Lander ES (2014). Searching for missing heritability: designing rare variant association studies. Proc Natl Acad Sci U S A.

[61.•] Agarwala V, Flannick J, Sunyaev S, Go TDC, Altshuler D (2013). Evaluating empirical bounds on complex disease genetic architecture. Nat Genet.

[62.•] Jun G, Manning A, Almeida M, Zawistowski M, Wood AR, Teslovich TM, Fuchsberger C, Feng S, Cingolani P, Gaulton KJ, Dyer T, Blackwell TW, Chen H, Chines PS, Choi S, Churchhouse C, Fontanillas P, King R, Lee SY, Lincoln SE, Trubetskoy V, DePristo M, Fingerlin T, Grossman R, Grundstad J, Heath A, Kim J, Kim YJ, Laramie J, Lee J, Li H, Liu X, Livne O, Locke AE, Maller J, Mazur A, Morris AP, Pollin TI, Ragona D, Reich D, Rivas MA, Scott LJ, Sim X, Tearle RG, Teo YY, Williams AL, Zöllner S, Curran JE, Peralta J, Akolkar B, Bell GI, Burtt NP, Cox NJ, Florez JC, Hanis CL, McKeon C, Mohlke KL, Seielstad M, Wilson JG, Atzmon G, Below JE, Dupuis J, Nicolae DL, Lehman D, Park T, Won S, Sladek R, Altshuler D, McCarthy MI, Duggirala R, Boehnke M, Frayling TM, Abecasis GR, Blangero J (2017). Evaluating the contribution of rare variants to type 2 diabetes and related traits using pedigrees. Proc Natl Acad Sci U S A.

[63.••] Mahajan A, Wessel J, Willems SM, Zhao W, Robertson NR, Chu AY (2018). Refining the accuracy of validated target identification through coding variant fine-mapping in type 2 diabetes. Nat Genet.

[64.••] Mahajan A, Taliun D, Thurner M, Robertson NR, Torres JM, Rayner NW (2018). Fine-mapping type 2 diabetes loci to single-variant resolution using high-density imputation and islet-specific epigenome maps. Nat Genet.

[65.••] Flannick J, Mercader JM, Fuchsberger C, Udler MS, Mahajan A, Wessel J, et al. Genetic discovery and translational decision support from exome sequencing of 20,791 type 2 diabetes cases and 24,440 controls from five ancestries. bioRxiv. 2018. 10.1101/371450. **This paper is the largest T2D exome sequencing study to date, demonstrating evidence for pervasive rare variant T2D gene-level signals but showing them to contribute minimally to T2D heritability**.

[66] Finucane HK, Bulik-Sullivan B, Gusev A, Trynka G, Reshef Y, Loh PR (2015). Partitioning heritability by functional annotation using genome-wide association summary statistics. Nat Genet.

[67] Sveinbjornsson G, Albrechtsen A, Zink F, Gudjonsson SA, Oddson A, Masson G (2016). Weighting sequence variants based on their annotation increases power of whole-genome association studies. Nat Genet.

[68] Altshuler D, Hirschhorn JN, Klannemark M, Lindgren CM, Vohl MC, Nemesh J, Lane CR, Schaffner SF, Bolk S, Brewer C, Tuomi T, Gaudet D, Hudson TJ, Daly M, Groop L, Lander ES (2000). The common PPARgamma Pro12Ala polymorphism is associated with decreased risk of type 2 diabetes. Nat Genet.

[69] Claussnitzer M, Dankel SN, Kim KH, Quon G, Meuleman W, Haugen C, Glunk V, Sousa IS, Beaudry JL, Puviindran V, Abdennur NA, Liu J, Svensson PA, Hsu YH, Drucker DJ, Mellgren G, Hui CC, Hauner H, Kellis M (2015). FTO obesity variant circuitry and adipocyte browning in humans. N Engl J Med.

[70] Flannick J, Florez JC (2016). Type 2 diabetes: genetic data sharing to advance complex disease research. Nat Rev Genet.

[71] Sladek R, Rocheleau G, Rung J, Dina C, Shen L, Serre D, Boutin P, Vincent D, Belisle A, Hadjadj S, Balkau B, Heude B, Charpentier G, Hudson TJ, Montpetit A, Pshezhetsky AV, Prentki M, Posner BI, Balding DJ, Meyre D, Polychronakos C, Froguel P (2007). A genome-wide association study identifies novel risk loci for type 2 diabetes. Nature..

[72] Rutter GA (2010). Think zinc: new roles for zinc in the control of insulin secretion. Islets..

[73] Nicolson TJ, Bellomo EA, Wijesekara N, Loder MK, Baldwin JM, Gyulkhandanyan AV, Koshkin V, Tarasov AI, Carzaniga R, Kronenberger K, Taneja TK, da Silva Xavier G, Libert S, Froguel P, Scharfmann R, Stetsyuk V, Ravassard P, Parker H, Gribble FM, Reimann F, Sladek R, Hughes SJ, Johnson PRV, Masseboeuf M, Burcelin R, Baldwin SA, Liu M, Lara-Lemus R, Arvan P, Schuit FC, Wheeler MB, Chimienti F, Rutter GA (2009). Insulin storage and glucose homeostasis in mice null for the granule zinc transporter ZnT8 and studies of the type 2 diabetes-associated variants. Diabetes..

[74] Pound LD, Sarkar SA, Ustione A, Dadi PK, Shadoan MK, Lee CE, Walters JA, Shiota M, McGuinness OP, Jacobson DA, Piston DW, Hutton JC, Powell DR, O’Brien RM (2012). The physiological effects of deleting the mouse SLC30A8 gene encoding zinc transporter-8 are influenced by gender and genetic background. PLoS One.

[75] Pound LD, Sarkar SA, Benninger RK, Wang Y, Suwanichkul A, Shadoan MK (2009). Deletion of the mouse Slc30a8 gene encoding zinc transporter-8 results in impaired insulin secretion. Biochem J.

[76] Wijesekara N, Dai FF, Hardy AB, Giglou PR, Bhattacharjee A, Koshkin V, Chimienti F, Gaisano HY, Rutter GA, Wheeler MB (2010). Beta cell-specific Znt8 deletion in mice causes marked defects in insulin processing, crystallisation and secretion. Diabetologia..

[77] Lemaire K, Ravier MA, Schraenen A, Creemers JW, Van de Plas R, Granvik M (2009). Insulin crystallization depends on zinc transporter ZnT8 expression, but is not required for normal glucose homeostasis in mice. Proc Natl Acad Sci U S A.

[78.•] Kleiner S, Gomez D, Megra B, Na E, Bhavsar R, Cavino K, Xin Y, Rojas J, Dominguez-Gutierrez G, Zambrowicz B, Carrat G, Chabosseau P, Hu M, Murphy AJ, Yancopoulos GD, Rutter GA, Gromada J (2018). Mice harboring the human SLC30A8 R138X loss-of-function mutation have increased insulin secretory capacity. Proc Natl Acad Sci U S A.

[79] Pal A, Barber TM, Van de Bunt M, Rudge SA, Zhang Q, Lachlan KL (2012). PTEN mutations as a cause of constitutive insulin sensitivity and obesity. N Engl J Med.

[80] Estrada K, Aukrust I, Bjorkhaug L, Burtt NP, Mercader JM, Consortium STD (2014). Association of a low-frequency variant in HNF1A with type 2 diabetes in a Latino population. Jama..

[81.•] Majithia AR, Tsuda B, Agostini M, Gnanapradeepan K, Rice R, Peloso G (2016). Prospective functional classification of all possible missense variants in PPARG. Nat Genet.

[82] Starita LM, Ahituv N, Dunham MJ, Kitzman JO, Roth FP, Seelig G, Shendure J, Fowler DM (2017). Variant interpretation: functional assays to the rescue. Am J Hum Genet.

[83] Findlay GM, Daza RM, Martin B, Zhang MD, Leith AP, Gasperini M, Janizek JD, Huang X, Starita LM, Shendure J (2018). Accurate classification of BRCA1 variants with saturation genome editing. Nature..

